# Medical examination powers miR-194-5p as a biomarker for postmenopausal osteoporosis

**DOI:** 10.1038/s41598-017-17075-w

**Published:** 2017-12-01

**Authors:** Haifeng Ding, Jia Meng, Wei Zhang, Zhangming Li, Wenjing Li, Mingming Zhang, Ying Fan, Qiujun Wang, Yina Zhang, Lihong Jiang, Wenliang Zhu

**Affiliations:** 1 Department of Geriatrics, the Second Affiliated Hospital of Harbin Medical University, 246 Xuefu Road, Harbin, 150001 China; 2 Department of Endocrinology, the First Hospital of Qiqihar, 30 Park Road, Qiqihar, 161005 China; 3 Department of Pharmacy, Guangdong Hospital of Integrated Chinese and Western Medicine, 16 Guichengnanwu Road, Foshan, 528200 China; 4 Institute of Clinical Pharmacology, the Second Affiliated Hospital of Harbin Medical University, 246 Xuefu Road, Harbin, 150001 China

## Abstract

An important attribute of microRNAs is their potential use as disease biomarkers. However, such applications may be restricted because of unsatisfactory performance of the microRNA of interest. Owing to moderate correlation with spine T-score, miR-194-5p was identified as a potential biomarker for postmenopausal osteoporosis. Here, we determined whether medical examination could improve its characteristic as a biomarker for postmenopausal osteoporosis. We recruited 230 postmenopausal Chinese women to measure circulating levels of miR-194-5p, determine the spine bone status, and perform a 42-item medical examination. No obvious information redundancy was observed between miR-194-5p and any one item. However, on examining miR-194-5p alone, the sensitivity at fixed specificity of 0.9 (SE_SP=0.9_) was 0.27, implying poor identification of at-risk individuals. Model integration of the microRNA and multiple medical items strengthened this property; in addition, model complexity greatly contributed to performance improvement. Using a model composed of two artificial neural networks, the ability of miR-194-5p to identify at-risk individuals significantly improved (SE_SP=0.9_ = 0.54) when correlated with five medical items: weight, age, left ventricular end systolic diameter, alanine aminotransferase, and urine epithelial cell count. We present a feasible way to achieve a more accurate microRNA-based biomarker for a disease of interest.

## Introduction

MicroRNAs (miRNAs) are a superclass of single-stranded non-coding RNAs composed of approximately 22 nucleotides^1^. Abundant evidence has suggested that circulating miRNAs are potential and feasible candidates in the current large-scope discovery and screening of clinical biomarkers for human diseases^2–5^. Usually, two modes can be selected for a miRNA-based biomarker, single and panel modes. For example, miR-21 alone can be used as a single biomarker for chemoresistance in oesophageal squamous cell carcinoma^6^. This miRNA can also be as a constituent element in a panel biomarker for early diagnosis of prostate cancer in combination with another miRNA (miR-375) and a non-miRNA indicator prostate-specific antigen^7^. Such flexible configurations and combinations have gradually made miRNAs potentially universal biomarkers for human diseases, although current miRNA measurements remain technically imperfect^8^.

Similarly to other diseases and pathologies, it has been discovered that many miRNAs including miR-133a^9–11^, miR-194-5p^12^, and miR-442a^13,14^ could be used as potential biomarkers for diagnosis and fracture risk prediction in postmenopausal osteoporosis, a high-incidence disease in the population of postmenopausal women^15^. The previously mentioned miRNA biomarkers were the optimal solution for postmenopausal osteoporosis among hundreds of candidate miRNAs using high-throughput screening tools such as miRNA microarrays. Different miRNAs have been discovered independently via similar research methods, implying that the prediction effectiveness of a miRNA biomarker may vary between populations. This may limit its clinical application in a wider geographical or ethnic range. For instance, Wang and his co-researchers developed a five-miRNA panel biomarker for non-small cell lung cancer (NSCLC) detection by recruiting a cohort from China^16^. The area under the receiver operating characteristic (ROC) curve (AUC) was 0.952 to identify NSCLC among the cohort, suggesting excellent performance. However, using the same biomarker, the AUC declined to 0.823 in a cohort from America, indicating minimized function for cancer detection. Another example is miR-652-3p, which was discovered as potential biomarker for breast cancer (BC) in a cohort of patients from Italy with an AUC of 0.83^17^. However, its performance markedly declined in a cohort of BC patients from America where an AUC of only 0.69 was obtained. It is important to highlight that these discrepancies are not an issue of the measurement technology, as previously discussed^8^.

To solve this problem, we propose a feasible way to improve the biomarker attribute of a miRNA by integrating it with medical examination items into a digital index using suitable mathematical modelling tools. To prove this hypothesis, miR-194-5p was measured in a cohort of 230 postmenopausal Chinese women. In addition, a 42-item medical examination was implemented. Our previous study demonstrated that the circulating expression levels of miR-194-5p were correlated to a decline in the spine bone mass density (BMD) in postmenopausal Chinese women^13^. Two machine-learning methods were attempted to obtain a better integration effect. The first method is a multiple linear regression (MLR), which has been widely used to develop panel miRNA biomarkers. Artificial neural network (ANN) has also been widely used in various areas of medicine. Medical application of ANN in medicine facilitates disease risk detection and medical decision-making^18–20^. In summary, the present study presents an integration-based attribute enhancement strategy for improving existing miRNA biomarkers. The feasibility of the strategy lies in that it depends only on routine clinical examination. We propose that such an effort could facilitate the application of circulating miRNAs as biomarkers in a clinical setting.

## Results

### Patients

A cohort of 230 postmenopausal Chinese women was recruited from Harbin as participants. Circulating levels of miR-194-5p were measured, spine health status, and a medical evaluation composed of 42 examination items (EIs) was determined for each participant. Dataset 1 presents all the raw data for all the participants. According to the spine L1–L4 T-score measurement, 92 participants (40.0%) had normal bone mass, 79 (34.3%) had been diagnosed with osteopenia, and 59 (25.7%) suffered from osteoporosis. Our survey results revealed that approximately three of every five postmenopausal Chinese women might be experiencing severe bone loss or had a predictable risk tendency for accelerated bone loss. Furthermore, an independent cohort of 30 postmenopausal Chinese women was recruited from Qiqihar, which is located ~360 kilometres northwest of Harbin. Dataset 2 presents the raw data for each of the 30 participants. From these, 12 participants (40.0%) had normal bone mass, 8 (26.7%) were diagnosed with osteopenia, and 10 (33.3%) suffered from osteoporosis.

### Literature search

**Figure 1:**
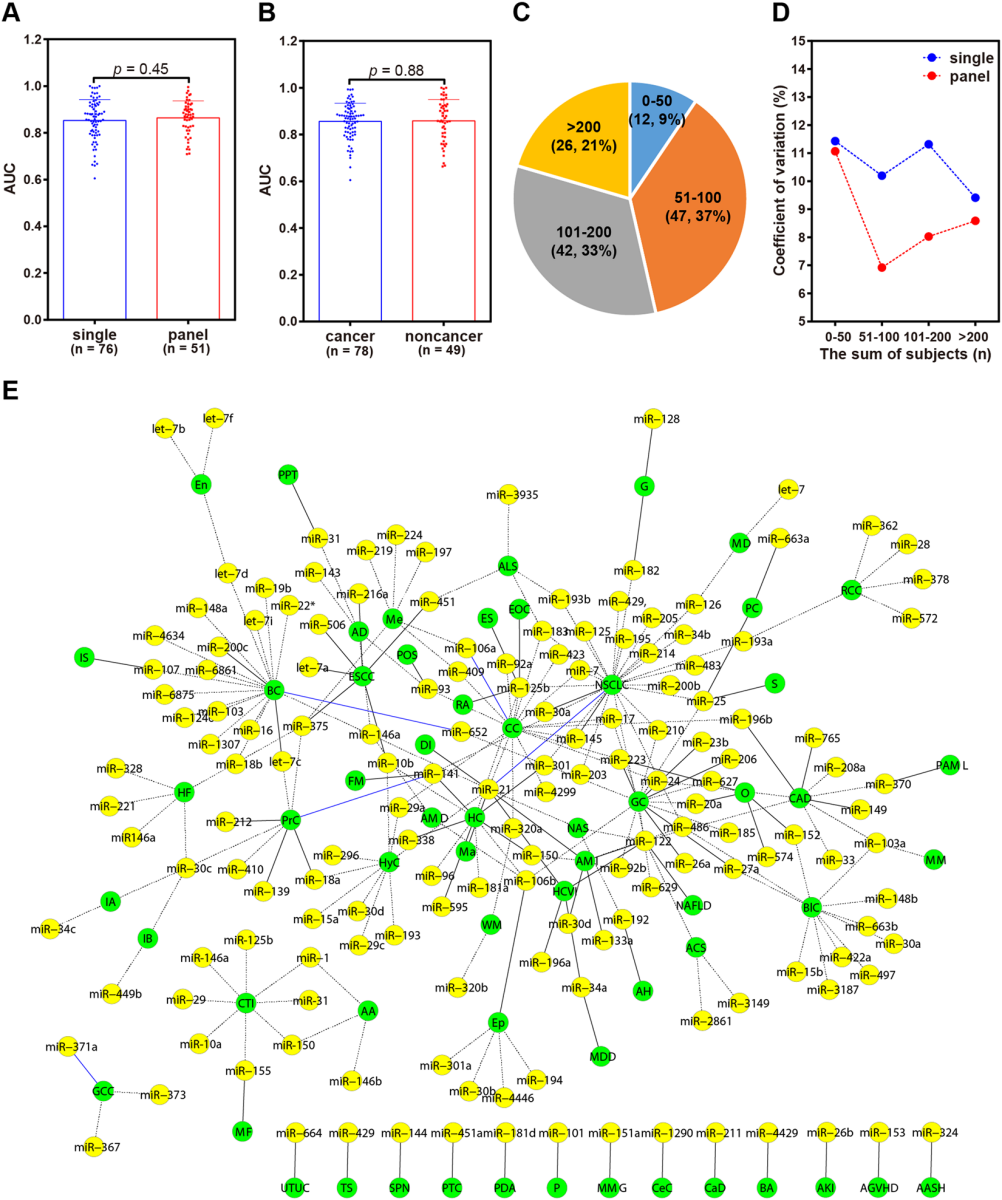
Literature-reported miRNA biomarkers for human diseases. Comparison of the recognition effects for high-risk subjects using single miRNA biomarkers and panel miRNA biomarkers (**A**) or from the aspect of disease category (**B**). A scatter plot was used and the data expressed as mean ± SD. Percentage distribution of studies (**C**) and coefficient of variation of AUCs (**D**) as the number of subjects was concerned. (**E**) Illustration of all the miRNA-disease associations in a network. The black solid-line and black dot-line indicate that a miRNA is used as a single biomarker or as an element for a panel biomarker with other miRNAs or non-miRNA markers, respectively; blue solid-line represents that a miRNA can be applied as a single biomarker and also used as a biomarker panel element. Only disease abbreviations are shown in green nodes for human diseases.

We conducted a literature search for potential miRNA biomarkers. According to the specified filters, 127 articles were found; from these, a total of 160 miRNAs were investigated for their feasibility to be biomarkers in 59 human diseases (Dataset 3). Seventy-six of the 160 miRNAs were considered by researchers as single miRNA biomarkers. However, more miRNAs were used to construct panel miRNA biomarkers with or without non-miRNA indicators. We did not observe significant differences in performance between the two miRNA biomarker types (Fig. 1A). About 62% of the articles investigated and successfully revealed miRNA biomarkers for cancer diseases; however, only 38% of the articles explored the possibility of miRNAs as biomarkers for non-cancer diseases. There were no obvious differences in performance when the two disease categories were compared (Fig. 1B). Mainly owing to objective reasons, most of the studies (~79%) were conducted on small cohorts of less than 200 participants (Fig. 1C). We measured the degree of dispersion of AUC in cohorts of different sizes (Fig. 1D). It was observed that when a cohort of more than 50 subjects was recruited, the prediction effectiveness of miRNA panel biomarkers was more robust than that of a single miRNA biomarker, suggesting that the panel mode was superior to the single mode in the context of marker stability. Furthermore, we graphically illustrate the relationship between miRNAs and diseases in a network (Fig. 1E). A salient issue for single miRNA biomarkers was that a single miRNA was validated for more than one disease by different research teams. For example, miR-21 was identified to be a single miRNA biomarker for at least five diseases: acute myocardial infarction, dengue infection, hepatitis C virus infection, macrosomia, and NSCLC^21–25^. This would result in poor prediction specificity for an actual clinical setting. The same issue could be observed for other single miRNA biomarkers such as miR-25, miR-34a, miR-122, miR-125b, miR-141, and miR-223 (Dataset 3).

### BMD-correlated EIs

**Figure 2:**
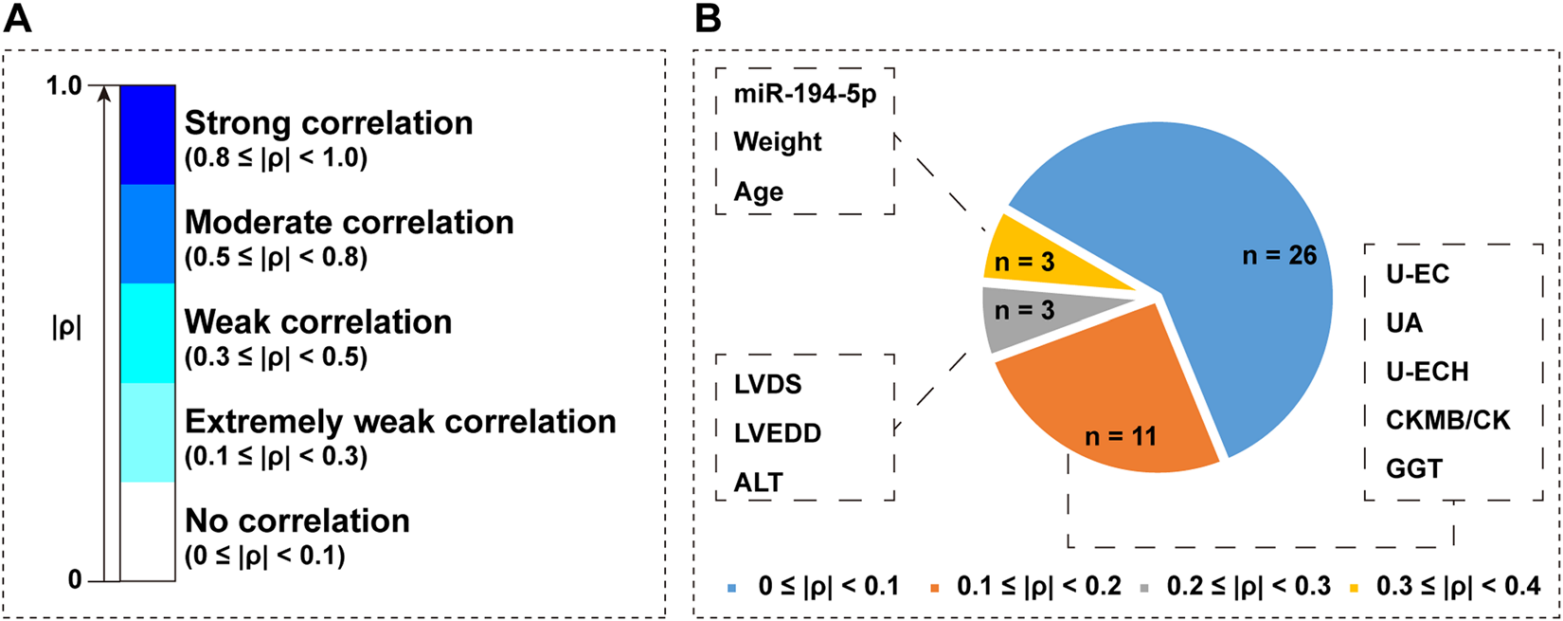
Correlation between the spine BMD and miR-194-5p or medical items. (**A**) Definition of the degree of correlation. (**B**) Distribution of miR-194-5p, and the 42 medical items according to Spearman’s rho values. Ten of the 42 medical items were highlighted because they passed the Spearman’s correlation test and were significantly correlated with the spine BMD (*p* < 0.01). LVDS: left ventricular end systolic diameter; LVEDD: left ventricular end diastolic diameter; ALT: alanine aminotransferase; U-EC: urine epithelial cell count; UA: uric acid; U-ECH: urine epithelial cells (high power field); CKMB/CK: creatine kinase isozyme/creatine kinase; GGT: γ-glutamyl transpeptidase.

A Spearman’s correlation test was performed to reveal BMD-correlated EIs among the 42 EIs described for the medical evaluation (Dataset 1). Not one EI was found to be moderately or strongly correlated with the spine BMD according to the standard definition of a degree of correlation (Fig. 2A). Together with miR-194-5p, age and weight were weakly correlated with the spine BMD (Fig. 2B). Comparatively, only an extremely weak correlation could be observed between the spine BMD and the other eight BMD-correlated EIs: left ventricular end systolic diameter (LVDS), left ventricular end diastolic diameter (LVEDD), alanine aminotransferase (ALT), urine epithelial cell count (U-EC), uric acid (UA), urine epithelial cells (high power field; U-ECH), creatine kinase isozyme/creatine kinase (CKMB/CK), and γ-glutamyl transpeptidase (GGT). Approximately 62% of the 42 EIs were found to have no meaningful correlation with the spine BMD (0 ≤ |ρ| < 0.1, ρ: Spearman’s rho).

### Integration optimization

**Figure 3:**
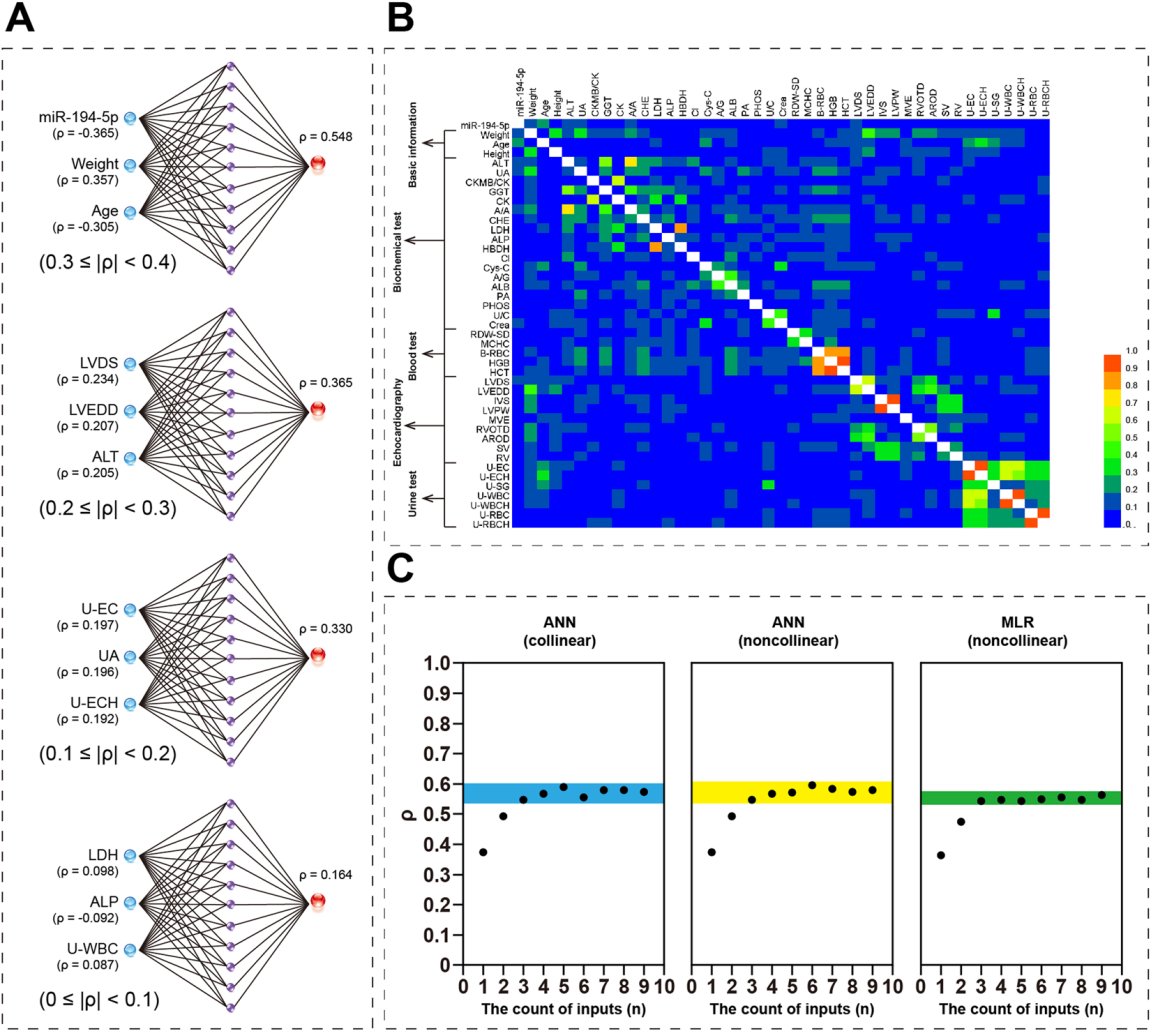
Model optimization for integration of miRNA and medical items. (**A**) The quality of model inputs determines the model integration effect. LVDS: left ventricular end systolic diameter; LVEDD: left ventricular end diastolic diameter; ALT: alanine aminotransferase; U-EC: urine epithelial cell count; UA: uric acid; U-ECH: urine epithelial cells (high power field); LDH: lactate dehydrogenase; ALP: alkaline phosphatase; U-WBC: urine white blood cell count. (**B**) Collinearity heatmap. With the exception of weight, age, and height, only the abbreviations are shown for the other medical items on the heatmap. (**C**) Influence of the number and the collinearity of model inputs and model type on the model integration effect. ‘Noncollinear’ means to exclude collinear inputs in the model, whereas ‘collinear’ indicates not to do so. ANN: artificial neural network; MLR: multivariate logistic regression.

It was proposed that integration of miR-194-5p with BMD-correlated EIs could strengthen the miRNA as a biomarker for postmenopausal osteoporosis. Before performing a formal model integration of miR-194-5p and BMD-correlated EIs, we investigated the potential influence of |ρ| values of single EIs, indicator collinearity, number of indicators to be integrated into the model, and model type on the final effect of model integration. It was found that indicators of higher |ρ| values resulted in better model integration than those of lower |ρ| values (Fig. 3A). Although no obvious collinearity existed between miR-194-5p and EIs, a remarkable collinearity could be found among EIs (Fig. 3B). For instance, a |ρ| value of 0.63 was calculated for the two echocardiography indicators, LVDS and LVEDD, both of which were identified as BMD-correlated EIs. By increasing the quantity of indicators to be integrated into the model, a higher |ρ| value could be obtained for the model output; however, this effect was decreased when the number of integrated EIs was continuously increased (Fig. 3C). A stable platform of |ρ| values appeared. Considering the issue of indicators, collinearity slightly improved the integration effect as the |ρ| value increased by 1.2%, from 0.589 to 0.596. Furthermore, it was observed that replacing the ANN model with a MLR model damaged such an effect. Eventually, by co-considering the above influencing factors, an optimal radial basis function (RBF)-ANN model was obtained, in which miR-194-5p and five BMD-correlated EIs (age, weight, LVDS, ALT, and U-EC) were used as inputs. The model was named ANN I in this study.

### Construction of derivative artificial neural network (DANN)

**Figure 4:**
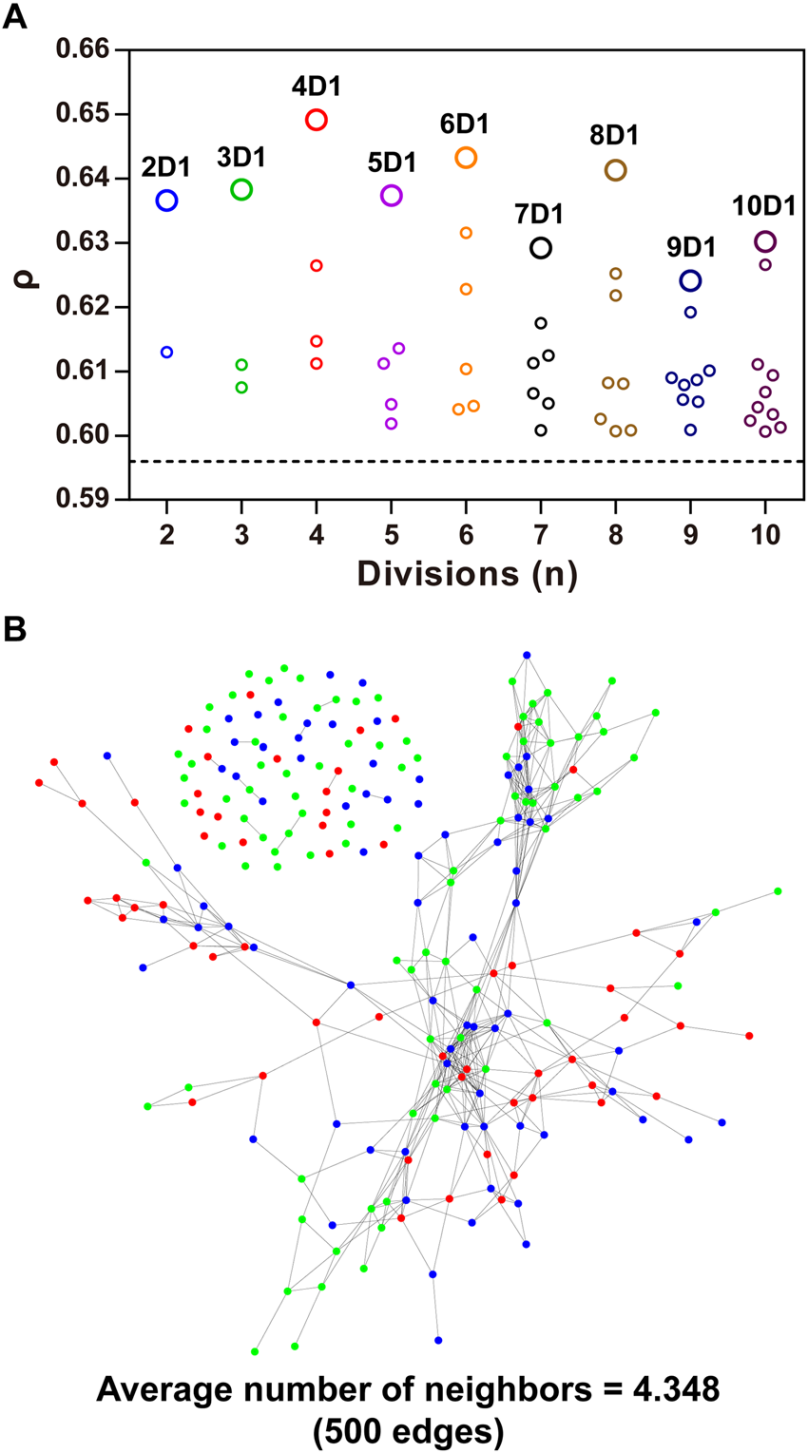
Patient division optimization to construct ANN II. (**A**) Four divisions of the patients’ lead to the strongest correlation between the ANN II outputs and the spine BMDs. (**B**) Patient-patient similarity network. Dots represent patient and edges indicate two similar patients (Euclidean distance < 0.198). Green, blue, and red represent normal bone mass, osteopenia and osteoporosis, respectively.

To explore whether model complexity could further improve the integration effect, we constructed a DANN (see Methods). For this purpose, the Euclidean distance between any two participants was calculated and the division size, *m*, was optimized to obtain the derivative information for each participant (see Methods). As Fig. 4A shows, the highest |ρ| value was obtained by ANN II when the division size was set to 4. Compared with ANN I, ANN II further increased the |ρ| value by 8.9%, from 0.596 to 0.649. Following the clustering algorithm proposed by Rodriguez and Laio^26^, we mapped all the participants into a participant-participant similarity network (PPSN) according to the calculated Euclidean distances among them (Fig. 4B). Two thirds of the participants were in a large cluster rather than in multiple smaller clusters. This finding was consistent with the fact that a small, rather than a bigger division size, led to an optimized ANN II (Fig. 4A).

### Evaluation of DANN as a panel biomarker for postmenopausal osteoporosis

**Figure 5:**
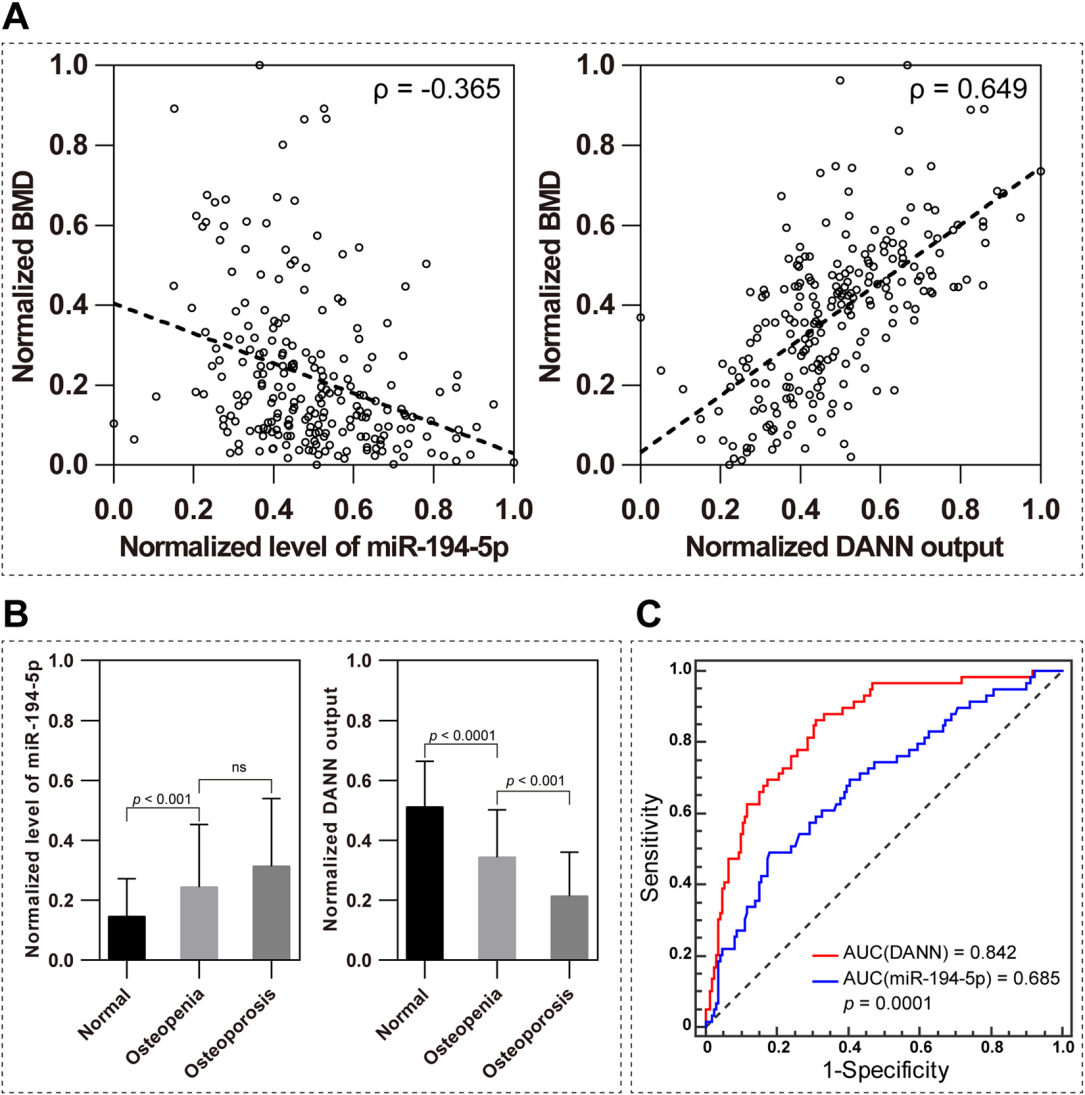
Comparison of single miRNA and DANN as a biomarker for postmenopausal osteoporosis. (**A**) Expression levels of miR-194-5p were weakly correlated with the spine BMDs whereas the DANN outputs constituted a moderate correlation with the spine BMDs. (**B**) Significant differences were observed between patients with osteopenia and those with osteoporosis using DANN instead of miR-194-5p. Data are expressed as mean ± SD. (**C**) Pairwise comparison of the receiver operating characteristic curves of miR-194-5p and DANN. Significant differences were found between the areas of the two curves. AUC: area under the curve; DANN: derivative artificial neural network.

We compared two models to assess the feasibility of miR-194-5p as a biomarker for postmenopausal osteoporosis, the single and the panel models. Circulating expression levels of miR-194-5p were weakly and negatively correlated with spine BMD (ρ = −0.365, Fig. 5A). By using miR-194-5p as a single biomarker for postmenopausal osteoporosis, we could not observe any obvious differences between participants with osteopenia and those suffering from osteoporosis (Fig. 5B). MiR-194-5p alone is not effective to distinguish between participants with osteoporosis, normal bone mass, or osteopenia (Fig. 5C); only an AUC of 0.685 could be expected. Sensitivity and specificity at the optimal cut-off point (SE_optimal_ and SP_optimal_) were 0.49 and 0.82, respectively; the sensitivity at fixed specificity of 0.9 (SE_SP=0.9_) was 0.27. In comparison, the biomarker attribute of miR-194-5p was remarkably strengthened by integration with the five BMD-correlated EIs (age, weight, LVDS, ALT, and U-EC) in DANN. A higher rank of correlation with the spine BMD was obtained for the DANN outputs (ρ = 0.649, Fig. 5A). As a result, we found significant differences between participants with osteopenia and those suffering from osteoporosis (*p* < 0.001, Fig. 5B). An AUC of 0.842 indicates good effectiveness of DANN to identify osteoporosis among the cohort (Fig. 5C). SE_optimal_ and SP_optimal_ were 0.86 and 0.69, respectively. The optimal cut-off point threshold was 0.38. SE_SP=0.9_ was 0.54, which doubled the value obtained using miR-194-5p as a single biomarker.

**Figure 6:**
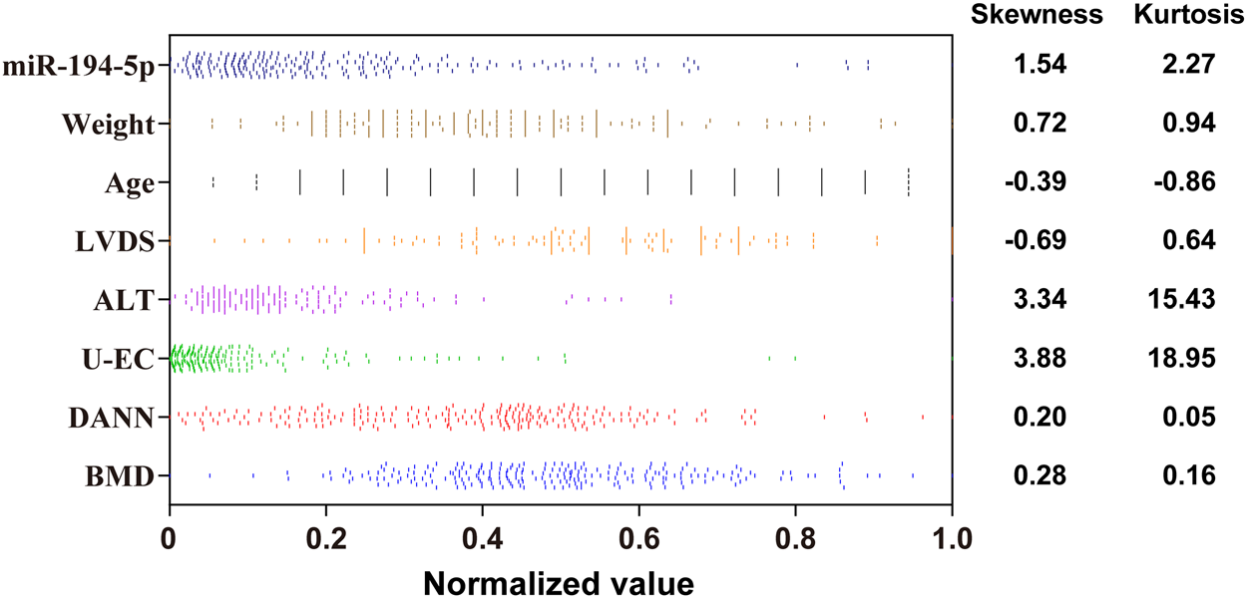
Scatterplots of the spine BMDs and the inputs and the outputs of DANN. The values of skewness and kurtosis of the corresponding scatterplots are shown on the right panel. LVDS: left ventricular end systolic diameter; ALT: alanine aminotransferase; U-EC: urine epithelial cell count; DANN: derivative artificial neural network.

Furthermore, the D’Agostino-Pearson omnibus normality test was applied to investigate the distribution characteristics of the expression values of miR-194-5p, the five BMD-correlated EIs, and the DANN output, along with the spine BMD. MiR-194-5p and the five BMD-correlated EIs did not meet the normal distribution (Fig. 6). Specifically, obvious skewness and kurtosis were observed for ALT and U-EC. However, the DANN output and the spine BMD were found to pass the D’Agostino-Pearson omnibus normality test, indicating normal distribution of their values without obvious skewness and kurtosis.

### Model validation

**Figure 7:**
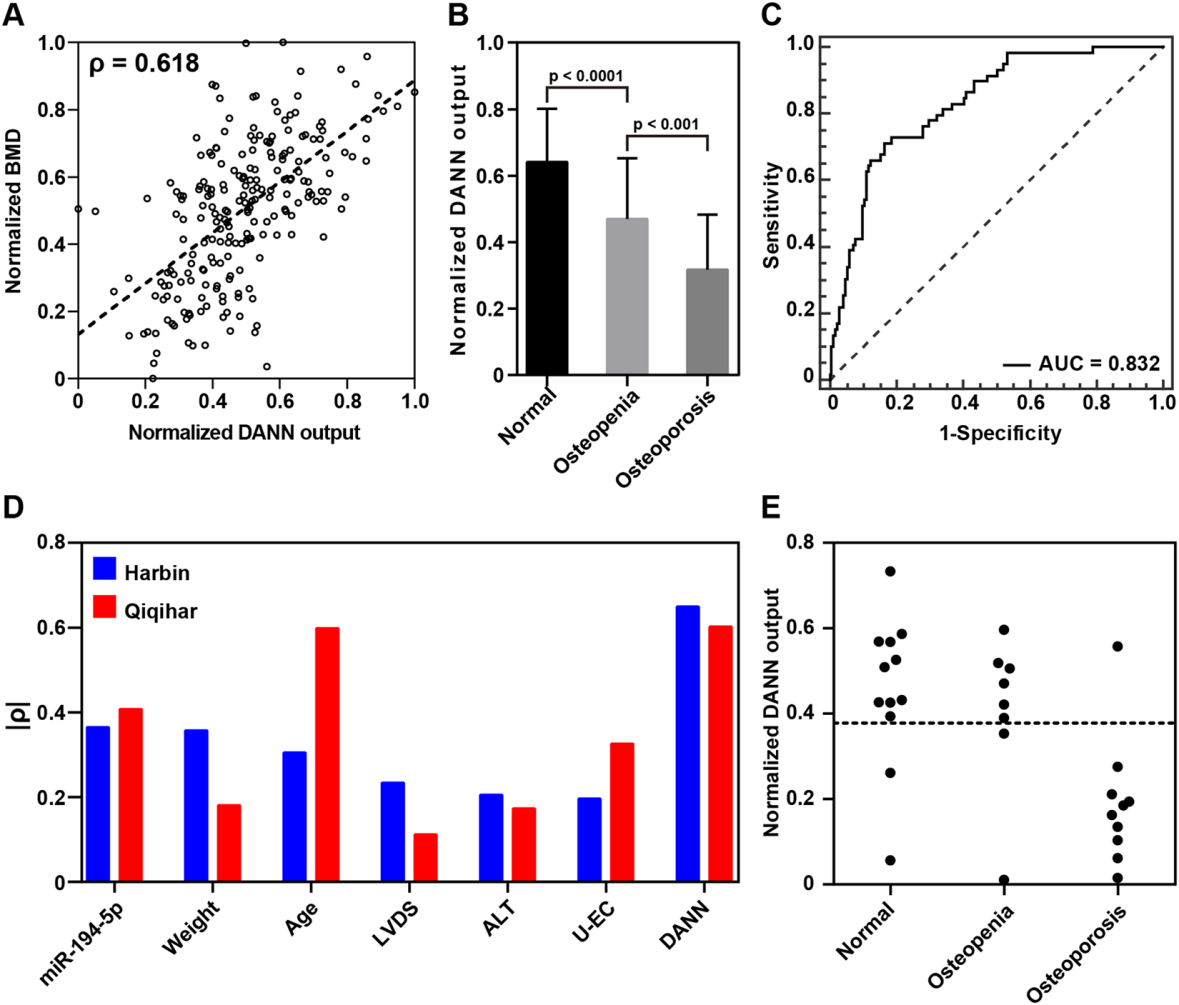
Results of the 10-fold cross-validation for DANN. (**A**) A Spearman’s rho of 0.618 indicates moderate correlation between the DANN outputs and the spine BMDs. (**B**) Significant differences were observed between patients with osteopenia and those with osteoporosis (*p* < 0.001). Data are expressed as mean ± SD. (**C**) An area under the curve of 0.832 suggests that DANN is effective for identifying at-risk individuals among a population of postmenopausal women. (**D**) Correlation coefficients between BMD and the expression values of miR-194-5p or EIs. (**E**) Scatter plot of the normalized NNC outputs for the participants recruited from Qiqihar. LVDS: left ventricular end systolic diameter; ALT: alanine aminotransferase; U-EC: urine epithelial cell count; DANN: derivative artificial neural network.

Two validation methods were used for the model validation of DANN. A holdout cross-validation method was used for assessing the generalization ability of ANNs I and II. Correlation coefficients for the training set (R_Tr_) and the testing set (R_Te_) were 0.592 and 0.563, respectively, for ANN I and 0.632 and 0.610, respectively, for ANN II, indicating good generalization ability of the two models. Moreover, a 10-fold cross-validation method was applied to evaluate the overall performance of the whole model system. A ρ of 0.618 and obvious differences between participants with osteopenia and those with osteoporosis ensure the ability of DANN to distinguish participants with osteoporosis form participants with normal bone mass or osteopenia (Fig. 7A–C).

An independent cohort of 30 postmenopausal Chinese women was recruited from Qiqihar to evaluate the performance of DANN in identifying osteoporosis from a geography aspect. Except for age, miR-194-5p and the other four EIs were found to not be significantly correlated with the spine BMD, suggesting geographical differences (Fig. 7D). Nevertheless, we could still confirm a remarkable correlation between DANN output and the spine BMD (ρ = 0.649, *p* = 0.004, Fig. 7D). When the optimal cut-off point threshold of 0.38 was used as the criteria, DANN successfully identified osteoporosis from the cohort with an accuracy of 83.3% (Fig. 7E).

## Discussion

Discovery-based miRNA biomarker research has provided considerable evidence for the potential application of circulating miRNAs as disease biomarkers in clinical settings^27^. Although technical issues about miRNA measurements still exist and no official standard or guideline has been developed, it is still optimistically considered that the bench-to-bedside translation of miRNA biomarkers will become a reality^8,28^.

Our literature survey led to several important points about circulating miRNA biomarkers. (1) A circulating miRNA can be used as a biomarker for multiple diseases and vice versa. A possible explanation is the ‘many-to-one’ and ‘one-to-many’ relationships between miRNAs and target genes. Because miRNAs post-transcriptionally regulate hundreds of target genes, a single miRNA may be essentially involved in the development of many diseases^29^. Altered miRNA expression in the peripheral blood should be considered a consequent result. (2) The effectiveness of a single miRNA biomarker varies in different populations, although there is little evidence to support this hypothesis^16,17^. An actual consequence is that superior performance of a single miRNA biomarker in one region/country may become meaningless in another region/country. (3) Generally, the effectiveness shown by a miRNA panel biomarker is equivalent to that exhibited by single miRNA biomarker. However, an apparent advantage of a miRNA panel biomarker over single miRNA biomarker is stability, especially for large cohorts consisting of 50 subjects or more. Stability refers to a more consistent performance of a miRNA panel biomarker in different studies. Taken together, the present review of 125 clinical studies results in an opinion about mode optimization of miRNA biomarkers. Compared to the single mode of a miRNA, a panel mode (miRNA in combination with other miRNAs or non-miRNA indicators) should be given a priority to establish a biomarker for a given disease.

In our previous study, miR-194-5p was successfully identified as a potential biomarker for postmenopausal osteoporosis^12^. Expression levels of circulating miR-194-5p were moderately correlated with the spine T-score in a cohort of 84 postmenopausal Chinese women. A pathway analysis revealed that miR-194-5p might be involved in many osteoporosis-related signalling pathways such as the transforming growth factor-beta (TGF-beta) and Wnt signalling pathways^30^. This result was in line with experimental evidence demonstrating that miR-194-5p stimulated osteogenesis and inhibited adipogenesis in mesenchymal stem cells^31^. Consistent with our previous study^12^, we further validated the significant correlation between the expression levels of circulating miR-194-5p and the spine BMD, but in a larger cohort of 230 postmenopausal Chinese women. ROC analysis result revealed moderate efficacy of miR-194-5p to identify osteoporosis in the cohort (AUC = 0.685). Due to moderate efficacy of miR-194-5p, a miRNA panel biomarker was the only mode to be considered for our biomarker design. Forty-two routine EIs were included in the screening of suitable panel constitute elements. Eventually, five EIs (age, weight, LVDS, ALT, and U-EC) were screened and combined with miR-194-5p in the panel. The integration of miRNA and EIs remarkably improved the efficacy of miR-194-5p in identifying osteoporosis (AUC = 0.842). An external validation on an independent cohort confirmed good performance of the miR-194-5p panel biomarker to identify osteoporosis with an accuracy of 83.3%. Furthermore, this preliminary result suggested that the performance of the miR-194-5p panel biomarker is steady. In this study, an external validation cohort was recruited from another city in China, 360 kilometres away from city where the original study was performed. Although a significant correlation was not found between the spine BMD and several panel constitute elements, the miR-194-5p panel biomarker could accurately identify subjects at risk. This finding was in line with our previous study where we established a miRNA panel biomarker for nasopharyngeal cancer^32^. Even though one of the panel constitute elements, miR-93, was artificially given the same expression value for all the patients, the miRNA panel biomarker still worked.

Based on the results of our literature survey, almost all the studies chose the MLR model to deal with the data integration of multiple panel constitute elements (Dataset 3). Our results suggest that application of a more complex model was beneficial to effectively improve the ability of biomarkers to identify risk. It should be noted that such a practice to improve biomarker performance did not depend on increasing the number of constituent elements in the panel, but in establishing a model with a more complex architecture. In another attempt, we confirmed the value of this practice; a DANN-based integration with only four EIs could effectively predict pulmonary fibrosis risk in patients with rheumatoid arthritis^33^.

In conclusion, we established a novel miRNA panel biomarker for postmenopausal osteoporosis, where miR-194-5p and five routine EIs constituted the panel elements. A limitation in this study is that the endogenous control U6 is a suboptimal reference gene. Although no significant Ct-value difference among normal bone mass, osteopenia, and osteoporosis was found by us, U6 was observed to be instable during freeze-and-thaw cycles^34,35^. So far, no consensus has been made about suitable reference gene for the quantification of circulating miRNAs. Despite this, preliminary but promising data have been provided in the present study, which constitute the basis for future research to further explore the potential of miR-194-5p as miRNA biomarker for postmenopausal osteoporosis. In this study, we systematically compared a single miRNA biomarker with a miRNA panel biomarker. A clear proposition was that miRNA panel biomarker should be a priority in clinical applications for use in more regions and achieve a more stable performance. Furthermore, model complexity should not be ignored if better risk recognition is the goal. Nevertheless, more research is needed to confirm the above views.

## Methods

### Ethical statement

This study was approved by the Ethics Committee of Harbin Medical University (Approval number: 2014-R-020) and the Ethics Committee of the First Hospital of Qiqihar (Approval number: 2016005). The present study was carried out in accordance with the Declaration of Helsinki. Each participant signed a written informed consent and was informed of detailed information regarding the research project.

### Participants

A total of 230 Chinese women inpatients from the department of geriatrics of the second affiliated hospital of Harbin Medical University and 30 Chinese women inpatients from the department of Endocrinology of the First Hospital of Qiqihar were recruited in the present study. Participants ranged from 48 to 66 years and have been postmenopausal for at least 12 months. A dual energy X-ray absorptiometry scanner (Hologic Inc., Bedford, MA, USA) was applied to measure BMD and T-score for the spine (between L1 and L4, L1–L4) for each participant. All the participants were divided into three groups according to the T-score: normal bone mass group (T-score > −1.0), osteopenia group (T-score ≤ −1.0 and >−2.5), and osteoporosis group (T-score ≤ −2.5). No participant reported fracture history and underwent any systematic anti-osteoporotic treatment before this study. For the participants from Harbin, 42 EIs were retrieved from the hospital’s electronic system of medical records when the participants were discharged from hospital (Dataset 1). We categorized the 42 EIs into five categories: basic information (3 items), biochemical test (18 items), blood test (5 items), echocardiography (9 items), and urine test (7 items). For the participants from Qiqihar, five EIs (age, weight, LVDS, ALT, and U-EC) were retrieved from the hospital’s electronic system of medical records when the participants were discharged from hospital (Dataset 2).

### Blood sample collection and total RNA extraction

Five millilitres of whole blood were obtained from each participant. Each whole blood sample was independently lysed using a Red Blood Cell Lysis Solution (Beyotime, Shanghai, China) and centrifuged for 10 min at 450 × *g*. The TRIzol reagent (Invitrogen, Shanghai, China) was used to extract total RNA from the precipitate. The isolated RNA eluate was stored at −80 °C for future experiments. To ensure sample consistency and stability, the RNA extraction procedure was completed within 30 min after blood collection from each participant.

### Quantitative real-time polymerase chain reaction (qRT-PCR)

Following the qRT-PCR assay method established in our previous study^12^, we measured the expression levels of miR-194-5p in each RNA extraction sample. Cycle threshold (CT) values of the miRNA were normalized to U6 and calculated using the equation 2^−∆∆CT^, as previously described^10^.

### Literature-reported miRNA biomarkers

A PubMed literature search was performed to obtain an overview of the currently validated miRNA biomarkers for human diseases. MicroRNA, biomarker, and receiver operating characteristic were used as joint search term. The search range was limited to recently published English-written articles with free text availability (from 2015.1.1 to 2016.10.1). Only articles with definite miRNA blood markers were considered for information extraction; reviews, meta-analysis articles, and those exploring non-blood samples or non-human samples should be discarded. The experimental evidence of miRNA biomarkers (Dataset3) was independently extracted by two researchers (Z.L. and Wenliang Z.). Any disagreements were resolved by consensus.

### Identification of BMD-correlated EIs

Because not all the EIs passed the D’Agostino-Pearson omnibus normality test or complied with a Gaussian distribution, a Spearman’s correlation test was performed (instead of a Pearson’s correlation test) to evaluate whether a given EI was correlated with the spine BMD using GraphPad Prism version 6.0 (GraphPad Software, Inc., La Jolla, CA, USA). A significant correlation with the spine BMD was accepted only if |ρ| (the absolute value of Spearman’s rho) was more than 0.1 and *p* was less than 0.01. MiR-194-5p and EIs that were significantly correlated with the spine BMD were retained for model integration as BMD-correlated information. Furthermore, we checked the extent of collinearity among miR-194-5p and the 42 EIs and graphically displayed the result in a heatmap using the HemI software version 1.0.3.3^36^. Collinearity was determined if a |ρ| > 0.3 was calculated.

### Model integration of miR-194-5p and BMD-correlated EIs

For BMD-correlated EIs, the expression values of miR-194-5p and the bone mass parameter BMD were normalized into a 0 to 1 number before further model integration was performed as previously described^32^. The Intelligent Problem Solver (IPS) tool in the software STATISTICA Neural Networks (SNN, Release 4.0E; Statsoft, Tulsa, OK, USA) was applied to construct a RBF-ANN model to investigate the effect of miRNA and EIs integration on the spine BMD association. The RBF-ANN model was named as ANN I in this study. The holdout cross-validation method was applied for preliminary validation of the model, as IPS randomly divided all the participants into three sets (training set, verification set, and testing set) in a 2:1:1 ratio. Thus, one-quarter of the participants was not included in model building and was used for model testing. The IPS calculated correlation coefficients for the training set (R_Tr_) and the testing set (R_Te_). The two correlation coefficients measured the correlation between model output and spine BMD. Similar values of R_Tr_ and R_Te_ indicate good generalization ability of the model. For model type comparison, the SPSS statistical software version 19.0 (IBM Corp., New York city, NY, USA) was applied to integrate miR-194-5p and BMD-correlated EIs into a MLR model.

### Euclidean distance calculation and construction of PPSN

For any two participants, we calculated their Euclidean distance in a six-dimensional space, in cases where miR-194-5p and the five BMD-correlated EIs (age, weight, LVDS, ALT, and U-EC) were re-defined as space coordinates. Following the clustering algorithm proposed by Rodriguez and Laio^26^, a PPSN was built using the network visualization software Cytoscape v2.8.3 (Institute of Systems Biology, Seattle, WA, USA)^37^.

### Obtaining and model integration of derivative participant information

Construction of the ANN I model and calculation of the Euclidean distance between participants were used to obtain derivative information for each participant. In our study, four items of derivative information were obtained for a participant as follows: first, we divided the other participants (except her) into *m*, mutually exclusive divisions of nearly equal size, according to the magnitude of their distances to her in the six-dimensional space. Thus, the four items of derivative information would be the ANN I output of the participant, mean ANN I output of participants in a given division, mean Euclidean distance to the participants in the division, and actual proportion of osteoporosis among participants in the division. The four derivative information items were imported in a new RBF-ANN as inputs to predict the participants’ potential for osteoporosis risk, in the same way as the ANN I. In order to distinguish it from ANN I, the new ANN was named as ANN II.

The effect of different participant groupings was investigated on the performance of ANN II. In this study, the division size *m* was assigned a digital value ranging from 2 to 10. For example, if we divided patients into five divisions, we would obtain five candidates of ANN II. The Spearman’s rho calculation was then used to identify the best model as ANN II. As a whole, we named the system developed here, DANN, which consists of ANN I and II.

### Model validation and performance evaluation

For DANN, the 10-fold cross-validation method was used for model validation as previously described^38^. Briefly, all the participants were randomly divided into 10 mutually exclusive sets of nearly equal size. Next, nine were selected for model training and one was used for model validation. The above procedure was repeated 10 times to allow each of the 10 participants sets to be independently used for validation. To investigate whether DANN distinguished between participants with osteoporosis and those with normal bone mass or osteopenia, we performed ROC curve analysis on outputs of DANN using MedCalc version 15.8 (MedCalc, Mariakerke, Belgium).

Besides AUC, we also recorded the values of sensitivity and specificity at the optimal cut-off point (SE_optimal_ and SP_optimal_, respectively) and the value of sensitivity at fixed specificity of 0.9 (SE_SP=0.9_) for model performance evaluation.

### Data statistics

Mean ± standard deviation (SD) was used for data presentation in the present study. GraphPad Prism version 6.0 was applied to conduct the following statistical analyses: D’Agostino-Pearson omnibus normality test, Student’s *t*-test, Spearman’s correlation test, and One-Way ANOVA test. Additionally, the MedCalc version 15.8 was used to perform pairwise comparisons of ROC curves based on the methodology of DeLong *et al*.^39^. In this study, differences were considered statistically significant when *p* < 0.01.

### Data availability statement

All data generated or analyzed during this study are included in this published article (and its Supplementary Information files).

## Electronic supplementary material

Dataset 1

Dataset 2

Dataset 3
